# Why workforce health should have a place in UK care reform

**DOI:** 10.1177/01410768221090673

**Published:** 2022-04-05

**Authors:** Lara Shemtob, Kaveh Asanati

**Affiliations:** Department of Primary Care & Public Health, School of Public Health, Imperial College London, London W6 8RP, UK

The National Health Service (NHS) and social care services are becoming more of a continuum. The government has pledged to use the new Health and Social Care Levy to improve training and support in the care sector as well as bring an end to the high costs of care faced by those who need it.^1^ The care workforce is in desperate need of investment, and unlike the NHS, the sector lacks unifying guidance around workforce health standards, despite facing many of the same occupational hazards and risks: biological from infectious diseases, ergonomic from manual handling and psychological from emotionally demanding work.^2–6^ The devastating way COVID-19 hit care homes in the first wave of the pandemic demonstrates the need for more regulation in this area, though mandating the COVID-19 vaccine for care home workers before any other occupational group was a thunderbolt for the sector. The adult social care survey in December 2021 was completed by around 10,000 providers in residential and domiciliary settings. Over 70% reported increasing challenges in recruiting and retaining staff, and maintaining staff morale.^7^ Bringing social care workforce occupational health policy in line with the standards set for NHS workers could improve patient care and abate the recruitment crisis. Where are we now with the health and wellbeing of the care workforce and where do we need to get to?

## Social care workforce structure

The organisational structure of the social care workforce is heterogeneous compared to healthcare, with an estimated 17,700 different organisations providing care, 37% of which have four employees or fewer.^8^ There has been a 30%–40% shift in the workforce away from local authority to independent sector employment since 2009. In 2018, 78% of jobs in adult social care were with independent employers, and only 7% in local authorities and 6% in the NHS. Regulated professionals (such as occupational therapists and social workers) make up 5% of the workforce^8^ but are more likely to be in public sector roles (comprising 17% of the local authority workforce). Professional regulation sets out standards and representation while carers delivering care at the bottom of the organisational hierarchy are least protected.

## What does this mean for occupational health policy?

Occupational health (OH) service provision is voluntary under the UK legislative framework. Private domestic employment is excluded from the Health and Safety at Work Act 1974^9^ and is challenging to regulate and therefore where carers are most vulnerable to exploitation. Private providers used by the NHS and local authorities fall within the Act, where there is most scope for improvement in OH policy. Without top-down guidance for the care sector it is impossible to understand what is happening across 17,700 providers with respect to workforce health. Research by the Department for Work and Pensions gives us an indication of the level of coverage at different types of organisations.^10^ An estimated 51% of employees in the UK have access to OH. Provision is skewed towards the public sector and larger organisations. Coverage drops to 21% of employees at organisations with fewer than 50 employees. Thirty-five percent of employers cited cost as the main barrier to offering OH services to their employees while employers that offered OH services cited cost savings and business efficiencies as key reasons for doing so.^10,11^

The NHS is the largest employer in healthcare. OH policies produced by the government and Public Health England filter down to an organisational level, and OH coverage for workers is comprehensive.^12–15^ There is a need for unifying policy reaching carers working across social care in the same way. It is important that care sector reform includes both standards for coverage and a means to report and audit this across different sorts of providers. The government’s current approach is limited; pledging reform via funding filtering down through local authorities is not guaranteed to reach direct carers nor lead to change in workforce health policies.

## Why is OH relevant to social care reform?

The importance of baking OH into social care reform is twofold. First, the fragmented sector needs cohesive guidance around workforce health to protect staff and patients. Second, improving workforce wellbeing and strengthening the appeal of social care work will go some way to tackling the recruitment crisis.^7^

Just like healthcare workers, social care workers come into contact with vulnerable patients. Yet, unlike healthcare workers there is no national guidance around worker health, an example being the lack of an immunisation programme. This is necessary to protect both carers and patients. Shingles, for example, common in elderly and immunocompromised patients, could have consequences in a carer without varicella zoster virus immunity, and for other patients they care for. While guidance addresses this risk in healthcare workers, there is no such guidance for the care sector. Employers may all take different approaches here, and it is difficult to establish what is happening in 17,700 organisations.

Immunisation is just one aspect of needing to improve the infrastructure around workforce health in care, from the emotional burden of dealing with mentally unwell or distressed patients to the physical demands of personal care. Carers need avenues for support, standards for dealing with periods of workforce illness and policies tackling absenteeism and presenteeism.

Standards and support to help the health of the care workforce will provide a means of addressing low recruitment, retention and morale in the sector. Care providers cited poor hours and working conditions as a main reason for staff leaving.^7^ The public consultation on mandating the COVID-19 vaccine revealed the possibility of alienating the workforce through taking sudden top-down action,^16^ confirmed by 15% of residential providers reporting mandatory vaccination as the main reason for carers leaving the workforce.^7^ Instead of an abrupt approach, trust needs to be built between the government, employers and employees in the sector around an agenda of carer health and wellbeing. The national insurance hike funding the Health and Social Care Levy may have the opposite effect in causing some employers to reduce carer wages. With no carers unions or professional bodies^5^ and workforce health and wellbeing falling outside of the Care Quality Commission's remit, a national approach to standards and audit is necessary to protect carers and those they care for. There is a paucity of research on occupational hazards in care, and how best to control occupational risks. Given the workforce crisis in care, increasing demand and shift towards greater public sector funding for services with the Health and Social Care Levy, addressing workforce health and wellbeing should be a priority area for occupational health research. The Health and Social Care Levy should catalyse an opportunity to support a greater understanding of the unmet need of care workforce health and wellbeing, with the goal of informing policy change. The aim should be a top- down sector wide solution of guidance and audit that the full range of providers can work towards, that will reach the different silos within the fragmented care sector. Whether the funds raised will be used for meaningful investment in the workforce remains to be seen.

## Declarations

### Guarantor

LS.

## References

[1] Record £36 billion investment to reform NHS and Social Care. GOV.UK. See www.gov.uk/government/news/record-36-billion-investment-to-reform-nhs-and-social-care (last checked 9 September 2021).

[2] Nursing Home Hazards. Health and Safety Authority. See www.hsa.ie/eng/Your_Industry/Healthcare_Sector/Occupational_Hazards_in_Nursing_Homes/Nursing_Home_Hazards/https:/www.hsa.ie/eng/Your_Industry/Healthcare_Sector/Occupational_Hazards_in_Nursing_Homes/Nursing_Home_Hazards/ (last checked 5 March 2022).

[bibr3-01410768221090673] Franklin P. Hazardous! Occupational safety and health in the care economy during the pandemic. Social Europe. 2020. See https://socialeurope.eu/hazardous-occupational-safety-and-health-in-the-care-economy-during-the-pandemic (last checked 5 March 2022).

[bibr4-01410768221090673] Sensible risk assessment in care settings – HSE. See www.hse.gov.uk/healthservices/sensible-risk-assessment-care-settings.htm (last checked 5 March 2022).

[bibr5-01410768221090673] Unison. Health and Safety Guidelines for home care workers. 16385.pdf. See www.nashics.org/wp-content/uploads/2012/08/16385.pdf (last checked 7 September 2021).

[6] Grasmo SG Liaset IF Redzovic SE . Home care workers’ experiences of work conditions related to their occupational health: a qualitative study. BMC Health Serv Res 2021; 21: 962–962. 34521407 10.1186/s12913-021-06941-z PMC8438557

[7] Adult social care workforce survey: December 2021 report. GOV.UK. See www.gov.uk/government/statistics/adult-social-care-workforce-survey-december-2021/adult-social-care-workforce-survey-december-2021-report (last checked 5 March 2022).

[8] State of the adult social care workforce in England 2021. Skills for care, Workforce intelligence. See www.skillsforcare.org.uk/adult-social-care-workforce-data-old/Workforce-intelligence/documents/State-of-the-adult-social-care-sector/The-State-of-the-Adult-Social-Care-Sector-and-Workforce-2021.pdf (last checked 5 March 2022).

[9] Domiciliary care provided in people’s own homes. See www.hse.gov.uk/healthservices/domiciliary-care.htm (last checked 5 March 2022).

[10] DWP. Health and wellbeing at work: a survey of employees, June 2015. See https://assets.publishing.service.gov.uk/government/uploads/system/uploads/attachment_data/file/447127/rr901-health-and-wellbeing-at-work.pdf (last checked 5 March 2022).

[11] Occupational_health_the_value_proposition_0.pdf. See www.som.org.uk/sites/som.org.uk/files/Occupational_health_the_value_proposition_0.pdf (last checked 5 March 2022).

[12] Green-Book-Chapter-12.pdf. See https://assets.publishing.service.gov.uk/government/uploads/system/uploads/attachment_data/file/147882/Green-Book-Chapter-12.pdf (last checked 28 February 2022).

[bibr13-01410768221090673] NHS health and wellbeing framework | NHS Employers. See www.nhsemployers.org/publications/nhs-health-and-wellbeing-framework (last checked 28 February 2022).

[bibr14-01410768221090673] Commissioning occupational health services | NHS Employers. Seewww.nhsemployers.org/publications/commissioning-occupational-health-services (last checked 5 March 2022).

[15] Introducing NHS Health at Work – NHS Health at Work Network. See www.nhshealthatwork.co.uk/ (last checked 28 February 2022).

[16] Making vaccination a condition of deployment in older adult care homes. GOV.UK. See www.gov.uk/government/consultations/making-vaccination-a-condition-of-deployment-in-older-adult-care-homes/making-vaccination-a-condition-of-deployment-in-older-adult-care-homes (last checked 5 March 2022).

[17] Find a regulator. See www.professionalstandards.org.uk/what-we-do/our-work-with-regulators/find-a-regulator (last checked 7 September 2021).

